# The transjugation machinery of *Thermus thermophilus*: Identification of TdtA, an ATPase involved in DNA donation

**DOI:** 10.1371/journal.pgen.1006669

**Published:** 2017-03-10

**Authors:** Alba Blesa, Ignacio Baquedano, Nieves G. Quintáns, Carlos P. Mata, José R. Castón, José Berenguer

**Affiliations:** 1 Centro de Biología Molecular Severo Ochoa, Universidad Autónoma de Madrid -Consejo Superior de Investigaciones Científicas, Madrid, Spain; 2 Department of Structure of Macromolecules, Centro Nacional de Biotecnología (CNB-CSIC), Cantoblanco, Madrid, Spain; Universidad de Sevilla, SPAIN

## Abstract

In addition to natural competence, some *Thermus thermophilus* strains show a high rate of DNA transfer *via* direct cell-to-cell contact. The process is bidirectional and follows a two-step model where the donor cell actively pushes out DNA and the recipient cell employs the natural competence system to take up the DNA, in a hybrid transformation-dependent conjugation process (transjugation). While the DNA uptake machinery is well known as in other bacterial species that undergo transformation, the pushing step of transjugation remains to be characterized. Here we have searched for hypothetical DNA translocases putatively involved in the pushing step of transjugation. Among candidates encoded by *T*. *thermophilus* HB27, the TdtA protein was found to be required for DNA pushing but not for DNA pulling during transjugation, without affecting other cellular processes. Purified TdtA shows ATPase activity and oligomerizes as hexamers with a central opening that can accommodate double-stranded DNA. The *tdtA* gene was found to belong to a mobile 14 kbp-long DNA element inserted within the 3′ end of a tRNA gene, flanked by 47 bp direct repeats. The insertion also encoded a homolog of bacteriophage site-specific recombinases and actively self-excised from the chromosome at high frequency to form an apparently non-replicative circular form. The insertion also encoded a type II restriction endonuclease and a NurA-like nuclease, whose activities were required for efficient transjugation. All these data support that TdtA belongs to a new type of Integrative and Conjugative Element which promotes the generalized and efficient transfer of genetic traits that could facilitate its co-selection among bacterial populations.

## Author summary

Transjugation is a new type of horizontal gene transfer process in which a donor cell pushes out genomic DNA upon cell contact and a recipient cell pulls this DNA inside by natural transformation. Here we describe TdtA, a DNA translocase of the pushing system of *T*. *thermophilus*, which is encoded within ICEth1, a new class of Integrative and Conjugative Element whose presence leads to generalized cell-to-cell transfer of any gene marker, circumventing the Argonaute surveillance system that controls access of extracellular DNA acquired by transformation.

## Introduction

Conjugation is the most widely distributed mechanism for horizontal sharing of genetic information in bacteria, favoring the dissemination of antibiotic resistance and spread of adaptive traits, providing an extraordinary source of intra- and interspecies genetic plasticity [1–4]. In well-known conjugation models, DNA transfer starts at a *cis*-acting site (o*riT*) and proceeds unidirectionally from the donor to a recipient cell that is in intimate contact. The process requires highly specialized machinery in the donor cell, including a complex type IV secretion system (T4SS) mobilization apparatus and a “relaxosome complex”. Relaxase is an enzyme that identifies a given *oriT* sequence, cuts its specifically, and remains covalently bound to the generated 5′ end. In the conjugation process, a tip protein of type 4 pilus binds to a compatible recipient cell, while further pilus retraction forms direct contacts through which the T4SS of the donor manages to generate a bridge to the cytoplasm of the recipient, allowing the recruitment and head-first translocation of the relaxase-ssDNA (single-stranded DNA) complex. In this classical conjugation model, the recipient cell remains passive, awaiting the arrival of DNA (reviewed by Lanka *et al* [5] and Zechner *et al* [6]).

Although also encoded by plasmids, the machinery for classical conjugation is frequently found within Integrative and Conjugative Elements (ICEs), mobile DNA elements that encode a phage-like site-specific integrase, which recognizes attachment sites (*att*) in the ICE and the genome, catalyzing its integration. Most frequently, the specific *att* site is located within the 3′ end of a tRNA gene, but *att* sites have also been observed in other genes. The *att* site (as much as 60 bp in length) duplicates as a consequence of insertion, its direct repeats allowing the identification of the ICE boundaries. Different stress conditions increase the frequency by which ICEs excise from the host genome, promoting self-encoded conjugative transfer to recipient cells. Some defective ICEs are also known to rely on conjugative plasmids, or even on other ICEs, to be mobilized. Also, defective plasmids can be mobilized by complementation with ICE-encoded proteins [7,8].

Alternative models for direct cell-to-cell DNA transfer have been reported in bacterial genera such as *Mycobacterium* and *Streptomyces* [9–11]. In *Mycobacterium*, a chromosome-encoded Distributive Conjugal Transfer (DCT) model [11,12] has been described, in which extensive fragments of non-contiguous chromosomal DNA are transferred in a unidirectional fashion from the donor to recipient cells, in the absence of known plasmids or ICEs [13]. Indeed, *cis*-acting sites show similar transfer efficiencies regardless of their chromosomal location, leading to extensive genomic complexity in the progeny, which have a mosaic-like architecture derived from both parental strains [11]. Some of these transconjugants can become donors in subsequent DCT [13].

In *Streptomyces* spp., conjugative plasmids encode a single DNA translocase (TraB), which is the only protein required for primary transfer of the plasmid to a recipient cell [14]. TraB is a large DNA translocase of the FtsK family that forms hexameric structures and catalyzes ATP-dependent double-stranded DNA (dsDNA) transfer to the recipient cell. The TraB protein also has a DNA recognition motif at its C-terminus which is responsible for identification of specific cis-acting locus of transfer (*clt*) sequences in the plasmid and, with lower frequency, in the chromosome. The conjugative plasmid also encodes a few Spd proteins which are required along with TraB for further intramycelial transfer across the septa, leading to rapid colonization of the whole mycelium [15].

In *Thermus thermophilus*, an unconventional conjugation-like mechanism has been described too. In this ancient phylum, cell contact-dependent DNA transfer occurs between completely isogenic strains, with no apparent order in the transfer and without the requirement for homologs of T4SS or relaxases. In *T*. *thermophilus* HB27, the process is even more efficient than transformation, with a 10-fold preference for transfer of genes encoded in the pTT27 megaplasmid compared to chromosomal genes. In this DNA transfer process, the natural competence machinery of the recipient cell plays an active role that is essential for the transfer, whereas competence is not required at all in the donor. For this reason, we proposed a two-step *“push-pull”* DNA model [16], where both mates play an active role. In this model, termed “transjugation” (from transformation-dependent conjugation), the donor cell actively pumps DNA *via* a hypothetical DNA translocase that is independent from the transformation machinery. In the second step, the competence apparatus actively pulls the DNA into the recipient cell.

In this work, we identified components of the pushing machinery by a combination of *in silico* screening and further genetic analysis. Through bioinformatics we identified putative orphan DNA translocases as targets for mutagenesis. Among the screened proteins, we identified TdtA as a major component required for DNA donation in transjugation. We determined that TdtA is a hexameric ATPase that binds to the membrane. We also found that the *tdtA* gene belongs to an active mobile genetic island integrated within a tRNA gene in what is the first evidence of the existence of an active new class of ICE-like elements in these phylogenetically ancient thermophiles.

## Results

### *T*. *thermophilus* HB27 encodes four putative DNA translocases

Because the hypothesized pushing step in transjugation requires DNA pumping from the donor cell, we performed a bioinformatics search across the genome of *T*. *thermophilus* HB27, looking for putative proteins harboring ATPase motifs (Walker A and B) and HAS-barrel motifs found in proteins of the FtsK-HerA family of ATPases, which are frequently involved in DNA translocation [17]. Four putative members of this protein family were found within the genome of *T*. *thermophilus* HB27, encoded by genes TTC0474 (FtsK, AAS80822, 867 aa), TTC0147 (AAS80495, 576 aa), TTC1430 (AAS81772, 611 aa), and TTC1879 (AAS82221, 568 aa). The putative amino acid sequence of the TTC0147 product shares 31% identity with that of TTC1879, whereas no significant sequence similarity was detected between the product of TTC1430 and that of TTC0147 or TTC1879.

To determine the roles of the putative proteins in DNA repair or as DNA translocases in segregation/conjugation, we attempted to generate knockout insertion mutants (*Δgene*::*kat*). For TTC0474, the gene encoding the likely homolog to FtsK, mutant recovery was unsuccessful despite the use of multiple different mutational strategies, suggesting its requirement for cell survival. In contrast, knockouts of the other three hypothetical genes were viable. As transjugation was not expected to affect viability or any other physiologically relevant cellular process, these mutants were subjected to UV-light and heat-shock, which increases mutation rates [18] and stress to discard those involved in cell survival. The results shown in Table 1 demonstrate significant (10 to 100-fold) differences between the TTC0147 and TTC1430 mutants compared to the wild type both for UV and heat shock survival assays (t-test; *p*-value< 0.001), whereas no difference was observed between ΔTTC1879::*kat* and the wild type in heat shock studies (t-tests; *p*-value: 0.678), and only marginal differences were found by the UV tests (t-tests; *p*-value: 0.038) (n = 8). On the other hand, transformation assays revealed a small decrease in efficiency with respect to the wild type for the mutants in TTC0147 and TTC1430, with the TTC1879::*kat* mutant not significantly affected.

**Table 1:** Phenotypic effects of deletion of FtsK-HerA homologs encoded by *T*. *thermophilus* HB27.

Strain	Survival to UV treatment	Survival to heat shock	Transformation efficiency
Wild type	1.07 ± 0.23	1.02 ± 0.86	18.51 ± 6.02 E-5
ΔTTC0147::*kat*	0.45 ± 0.03	0.02 ± 0.01	6.71 ± 2.47 E-5
ΔTTC1430::*kat*	0.02 ± 0.01	0.13 ± 0.04	9.02 ± 0.76 E-5
ΔTTC1879::*kat*	0.75 ± 0.04	0.87 ± 0.51	12.47 ± 4.02 E-5

Therefore, TTC0147 and TTC1430 seem to play some relevant role in cell physiology, likely in DNA repair after stress, whereas TTC1879 is dispensable, as could be expected for a transjugation-associated gene.

### TTC1879 is required for transjugation

**Fig 1:**
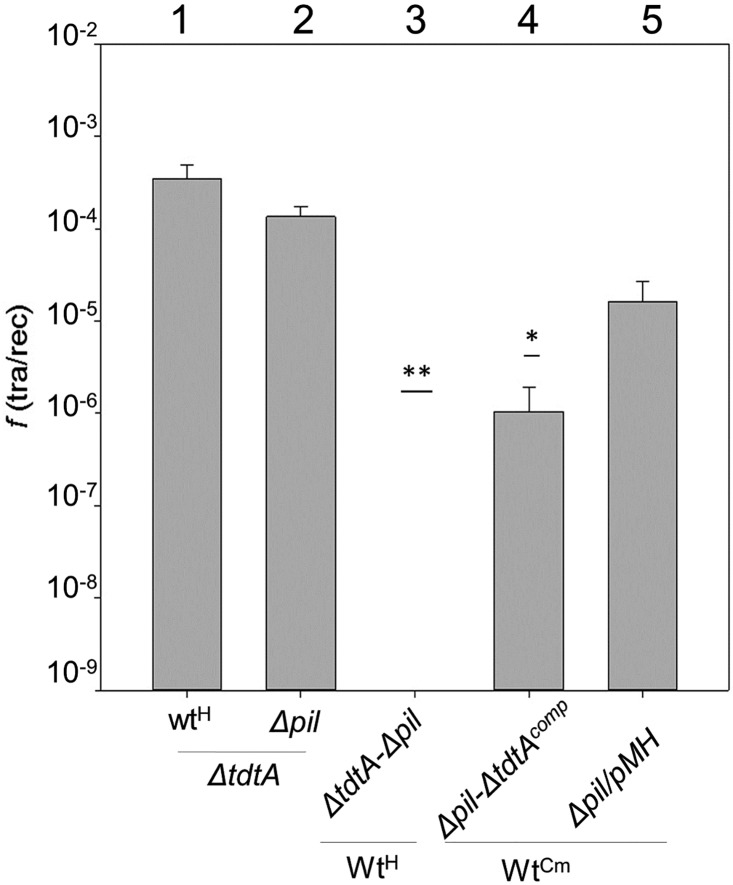
The product of TTC1879 (TdtA) is required for transjugation. Transfer frequencies are expressed as the ratio of transjugant: wild type CFU. Bars 1 and 2 correspond to matings between a *tdtA* mutant (*ΔtdtA*) and a Hyg^r^ wild type strain (wt^H^) (1) or a *ΔpilA4* competence mutant (*Δpil*) (2). Mating between the wt^H^ strain and a double *tdtA* and *ΔpilA4* mutant (*ΔtdtA-ΔpilA)* rendered no transjugants (3). The expression of TdtA from a plasmid in this double mutant (*Δpil-ΔtdtA*^*comp*^) allowed for the generation of transjugants in matings with a Cm^r^ wild type (wt^Cm^) (4). Control matings between the same wt^Cm^ strain and a single *ΔpilA4* mutant carrying the empty plasmid (*Δpil/pMH*) showed a 10-fold higher transjugation frequency (5). ANOVA tests showed significant differences among frequencies of transfer of all the derivatives plotted (*p*-value< 0.001) and *post-hoc* Holm Sidak tests proved that absence of *tdtA* has an effect on transjugation (n = 8). Asterisks indicate significant statistical differences compared to the wild type (*: p-value>0.05;**p-value<0.001).

To analyze the role of TTC1879 in DNA donation (pushing step) we introduced the ΔTTC1879::*kat* mutation into a *ΔpilA4* background, which is unable to serve as a recipient in transjugation assays [16], and mated it with a wild type strain labeled with hygromycin (Hyg) resistance. No transjugants were generated in this mating (Fig 1, bar n° 3), implying that the ΔTTC1879::*kat* mutant cannot donate DNA in transjugation. As expected from the bidirectionality of the transjugation process, mating assays between the single ΔTTC1879::*kat* mutant and the wild type produced transjugants resistant to kanamycin (Km) and Hyg at high frequencies (Fig 1, bar n° 1), illustrating that the ΔTTC1879::*kat* mutant can act as a recipient in transjugation. On the other hand, transjugation between a chloramphenicol (Cm)-resistant wild type strain and a ΔTTC1879::*kat-ΔpilA4* double mutant carrying a multicopy plasmid (pMHTTC1879, Hyg^r^) ectopically expressing the product of TTC1879, complemented the transjugation capability(Fig 1, bar n° 4). A control *pilA4* mutant (wild type for TTC1879) carrying an empty Hyg^r^ plasmid had transjugation frequencies about one order of magnitude higher (Fig 1, bar n° 5). On the other hand efficient transjugation was detected between ΔTTC1879::*kat* and *ΔpilA*::*hyg* single mutants (Fig 1, bar n° 2). As two strains defective in competence cannot mate [16,19], this result confirmed that the product of TTC1879 was not involved in natural competence, and provides evidence that TTC1879 is required for DNA donation in transjugation, and not for DNA acquisition. The product of TTC1879 was thus designated the Transjugation Donor Translocase A (TdtA).

**Fig 2:**
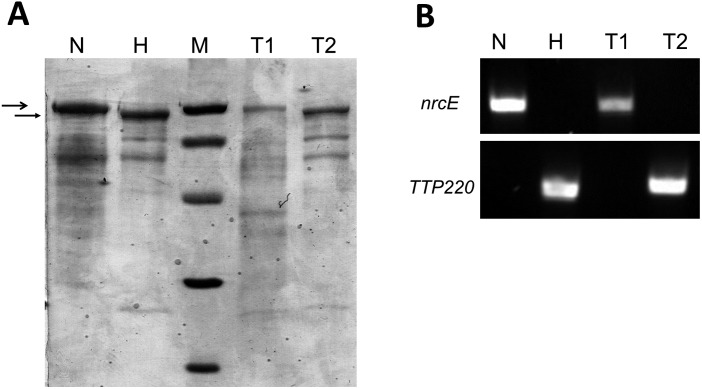
TdtA is required in the donor strain for transjugation. **(A)** SDS-PAGE electrophoresis showing membrane protein profiles. Patterns corresponding to the *ΔtdtA*::*kat* mutants derived from NAR1 (N) and HB27 (H) and the corresponding patterns after transjugation between NAR1-*ΔtdtA* and HB27-Wt (T1) and between HB27-*ΔtdtA* and NAR1-Wt (T2). Lane M shows protein molecular standards at 97.4, 66.2, 45, 31, and 21.5 kDa. Large and small arrowheads signal the S layer proteins of 100 kDa and 97 kDa corresponding to the NAR1 and HB27 strains, respectively, used as main strain identification marker. Note the similarities between lanes N and T1, and between lanes H and T2, supporting that transjugants derive from the respective *tdtA* mutant in the matings. **(B)** Agarose gel electrophoresis showing PCR amplicons of *nrcE* (upper panel), specific to the NAR1 strain, and *ttp0220* (lower panel), specific to the HB27 strain using DNA extracted from *ΔtdtA*::*kat* mutants derived from NAR1 (N) and HB27 (H), the transjugants pool from mating experiments between NAR1-*ΔtdtA* and HB27-Wt (T1), and the transjugants pool of the reciprocal mating between HB27-*ΔtdtA* and NAR1-Wt (T2). Oligonucleotide sequences are shown in S2 Table.

To further confirm that TdtA was required only for DNA donation in transjugation, PCR was used to screen different strains of *T*. *thermophilus*, finding a homolog with 100% sequence identity to TdtA in the nitrate-respiring NAR1 strain. This strain can be distinguished from HB27 both by PCR and by differences in the pattern of their membrane proteins, specifically size differences of S-layer proteins (SlpA) [20], which enables inference of the directionality of DNA transfer by parenthood analysis. The NAR1 and HB27 wild type strains transjugate efficiently in both directions [16]. To analyze the role of TdtA, a *ΔtdtA*::*kat* mutant was derived from NAR1 and used in transjugation assays with a Hyg^r^ derivative of the HB27 strain. The whole pool of transjugants from this mating experiment was harvested from the plates and analyzed to identify the parenthood. The membrane protein pattern of the transjugants was similar to that of NAR1, characterized by a 100 kDa SlpA protein (Fig 2A; lane N *vs*. lane T1), and PCR assays revealed the presence of the NAR1-specific *nrcE* gene but the absence of the HB27-specific *TTP220* gene (Fig 2B; lane N *vs*. lane T1). These results show that the NAR1 *ΔtdtA*::*kat* mutant serves as a recipient and not as a donor in transjugation. Conversely, transjugants from a mating between an HB27-derived *ΔtdtA*::*kat* mutant and a Hyg^r^ NAR1 wild type strain possessed the smaller SlpA protein of HB27 (Fig 2A; lane H *vs*. lane T2), the HB27-specific TTP220 gene, and lacked *nrcE* (Fig 2B; lane H *vs*. lane T2), indicating that the HB27 *ΔtdtA*::*kat* mutant can function only as recipient in transjugation and not as donor. These experiments provide solid evidence that the absence of TdtA impairs the ability of the cell to act as a donor in transjugation, but does not affect its ability to serve as recipient.

### TdtA is expressed from a 4-gene operon, and localizes to the cytoplasmic membrane

**Fig 3:**
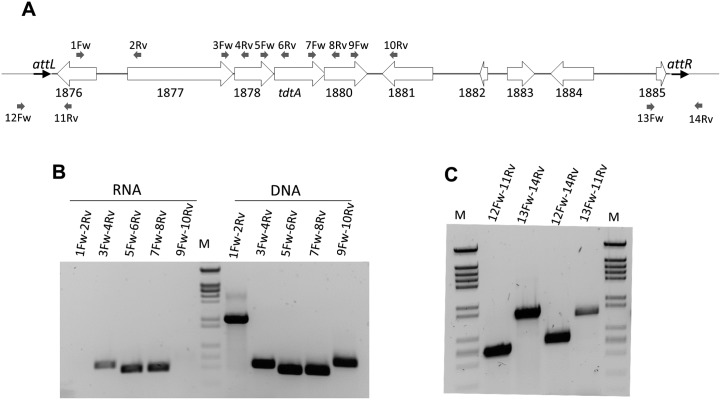
TdtA is encoded within an active ICE-like element. **(A)** Scheme showing the context of *tdtA* in the HB27 chromosome. White arrows represent ORFs encoded by the genes indicated underneath and are scaled proportionally to their size. The thick grey line is also proportional to the length of the whole ICEth1. Small grey arrows represent the relative position of primers employed in RT-PCR assays shown in panel B. **(B)** RT-PCR assays with the indicated primer pairs were conducted to amplify the intergenic regions between the genes TTC1877-1878-tdtA-1880. Parallel control PCRs performed on genomic DNA are shown at the right. **(C)** Agarose gel showing PCR amplification from genomic DNA of exponential cultures of *T*. *thermophilus* HB27 with the indicated primers to detect the ends of the ICEth1 in its integrated form (12Fw-11Rv and 13Fw-14Rv), the DNA scar produced by it excision (12Fw-14Rv), and the excised circular form (13Fw-11Rv).

Analysis of *tdtA*’s position within the genome of *T*. *thermophilus* HB27 is presented in Fig 3A. Upstream of *tdtA* is a gene that encodes a putative nuclease of the NurA family (TTC1878; 377 aa), which are frequently found immediately upstream of proteins from the FtsK-HerA superfamily. Further upstream, TTC1877 encodes a large protein (1106 aa) that is identical to Tth111II, a type IIG restriction endonuclease previously characterized from another strain of *T*. *thermophilus* [21]. Downstream from *tdtA*, TTC1880 encodes a hypothetical 410 aa protein of the N4-N6 methylase family (pfam 01555).

These four genes are separated by 4 (TTC1877-1878), 2 (TTC1878-*tdtA*), and -4 (*tdtA*-TTC1880, overlapping) bp, and all other nearby genes are encoded in the opposite orientation. Therefore, these four genes are likely co-transcribed from a promoter upstream of TTC1877. In fact, RT-PCRs spanning each intergenic region between TTC1877 and TTC1880 produced successful amplification from total mRNA of *T*. *thermophilus* HB27, whereas amplification failed using primers bridging the TTC1876-1877 and TTC1880-1881 intergenic regions (Fig 3B), thus confirming co-transcription of a four-gene operon.

**Fig 4:**
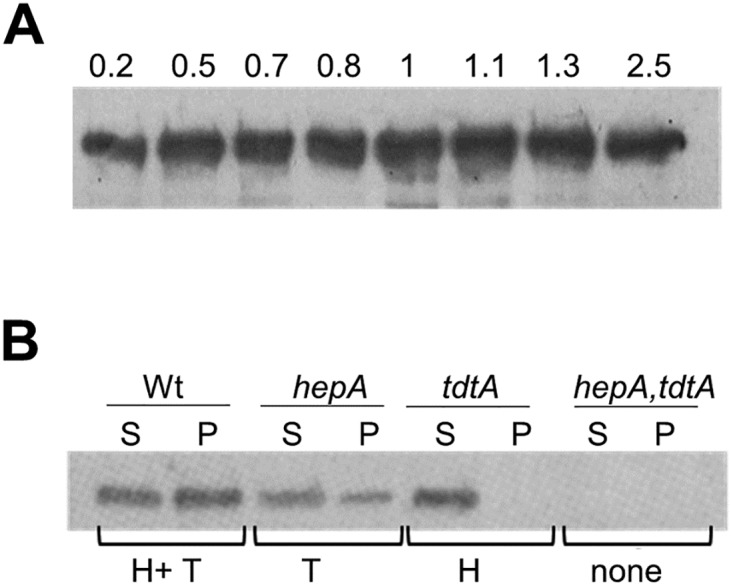
Expression and subcellular localization of TdtA. **(A)** The expression of the TdtA-YFP fusion from its native promoter in the chromosome was followed by western blot with anti-GFP antiserum throughout growth at 60°C. Identical cell mass was analyzed at the indicated optical densities at 550 nm **(B)** Western blot with an antiserum that cross-reacts with both TdtA and a HerA-like protein (product of TTC0147 baptized as HepA) was used to localize the proteins in soluble (S) and non-soluble (P) fractions from the following strains of *T*. *thermophilus* HB27: wild type (Wt), Δ*hepA*::*kat* (*hepA*), Δ*tdtA*::*kat* (*tdtA*) and Δ*tdtA*::*kat*, *hepA*::*hyg* (*hepA*,*tdtA*). The proteins detected in each case are indicated underneath: TdtA (T) and HepA (H).

To analyze the expression of the operon throughout the growth cycle, the TdtA protein was tagged with a thermostable fluorescent protein (TdtA-sYFP), allowing its detection by western blot. TdtA was detected at similar levels from the exponential through the stationary phase of growth (Fig 4A).

The role of TdtA in pushing DNA out of the cells could imply a connection to the membrane. To test this, a rabbit antiserum that cross-reacts with both TdtA and the product of TTC0147 (a putative HerA-like helicase) was employed. Proteins of the expected size (approximately 64 kDa for both proteins) were detected in both the soluble (S) and the cell envelope insoluble (P) fractions of the wild type strain, whereas no signal was detected in a *ΔtdtA*-TTC0147 double-deletion mutant (Fig 4B). In the TTC0147 single-deletion mutant, TdtA was detected in both the S and P fractions. In contrast, in a *ΔtdtA* context the TTC0147 product was detected only in the S fraction. Keeping in mind that part of the TdtA protein is found in the soluble fraction, and that its primary sequence does not contain a putative secretion signal, the most likely location for the TdtA protein is the cytoplasmic membrane.

#### The NurA-homolog and the Tth111II restriction enzyme encoded by the *tdtA* operon are required for efficient transjugation

**Fig 5:**
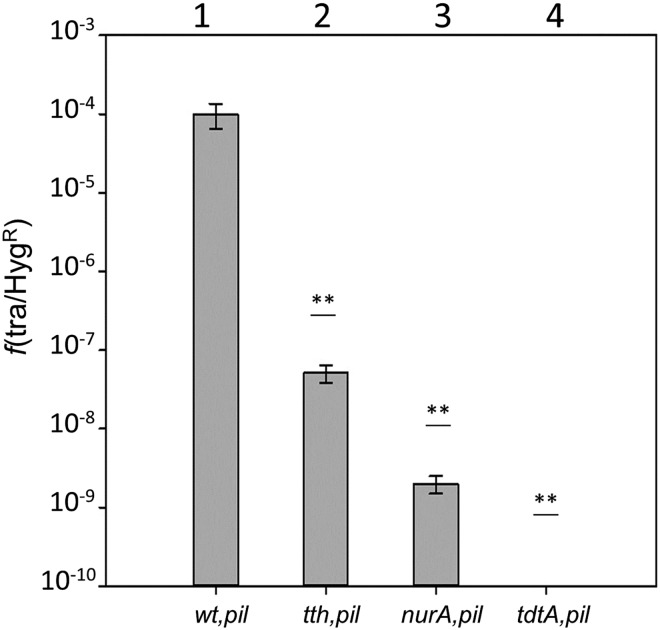
Effects of the NurA-homolog and Tth111II on transjugation. The frequencies of transjugation assays between *pilA* mutants labeled with kanamycin at the *gdh* (*wt*,*pil*) locus (1), or at the genes encoding Tth111II (*tth*,*pil*) (2), NurA-like (*nurA*,*pil*) (3) or TdtA (*tdtA*,*pilA*) (4) and a wild type strain labeled with Hyg^r^ are shown. Asterisks indicate significant statistical differences compared to the wild type (*p*-value<0.001) (n = 6).

The putative role in transjugation of the NurA-like protein and Tth111II restriction enzyme was analyzed using the same method as done previously for TdtA. In essence, *ΔpilA4* -TTC1877::*kat* and *ΔpilA4* -TTC1878::*kat* double mutants were used in mating experiments with a Hyg^r^ wild type. The results show that the strain lacking TTC1878 (*nurA*-like) suffered a decrease in transjugation efficiency of 4–5 orders of magnitude with respect to a *ΔpilA4* control strain labeled at another position in the chromosome (Fig 5, bars 1 vs. 2), whereas the mutant without the Tth111II restriction enzyme showed a decrease of around 3 orders of magnitude (Fig 5, bars 1 vs. 3). As expected, a *ΔpilA4-tdtA*::*kat* control rendered no detectable transjugants in mates with the wild type (Fig 5, bar 4). These data support that both genes somehow cooperate with TdtA to enhance the transjugation process.

#### TdtA is encoded within a new ICE

Upstream of the *tdtA* operon, the TTC1876 gene encodes a homolog to phage and ICE integrases of the XerC family (392 aa), while a putative DDE transposase (TTC1881, 542 aa) was found downstream. With the exception of this transposase (of which two more chromosomal copies exist), the whole region encompassing *tdtA* has a lower G+C content (58%) than the average of its genome (68%). Actually, the limits of this low G+C content region were defined by two 47-bp direct repeats (labeled *attL* and *attR* in Fig 3A) that are identical in sequence to the 3′ end of an isoleucine tRNA. The whole 14,857 bp region is absent from the closely related strain *T*. *therm*ophilus HB8.

As these properties are common to ICEs, the region was designated ICEth1. To determine whether ICEth1 was able to excise from the chromosome, PCR assays were used (Fig 3C); primer pairs 12Fw/11Rv and 13Fw/14Rv allowed amplification of the ends of ICEth1 in its integrated form (amplicons of 566 and 1,097 bp for the left and right sides, respectively), whereas primers 12Fw/14Rv produce a 646 bp amplicon only from genomes in which the ICEth1 had been excised, leaving the corresponding scar behind. Both the left and right arms of the integrated form and the scar were detected by PCR, showing that the ICEth1 had excised from the chromosome in a fraction of the cells in the culture. In addition, primers 13Fw/11Rv detected a 1,017 bp amplicon corresponding to the circularized form of ICEth1, albeit present at lower levels compared to the *attB* scar produced by its excision.

To quantify the frequency of this excision during growth in rich medium, the percentage loss of the Km^r^ phenotype was compared between the *ΔtdtA*::*kat* mutant and a non-excisable Km^r^ chromosomal marker (*Δgdh*::*kat*). Up to 8% of the *ΔtdtA*::*kat* colonies from exponential cultures (OD_600_ = 0.6) had lost the Km^r^ marker, whereas no loss was detected in the *Δgdh*::*kat* mutant under the same experimental conditions (p-value < 0.001). These data demonstrate that ICEth1 excises from the chromosome at high frequencies during exponential growth, in a TdtA-independent manner.

#### The presence of the ICEth1 enhances transjugation dramatically

**Fig 6:**
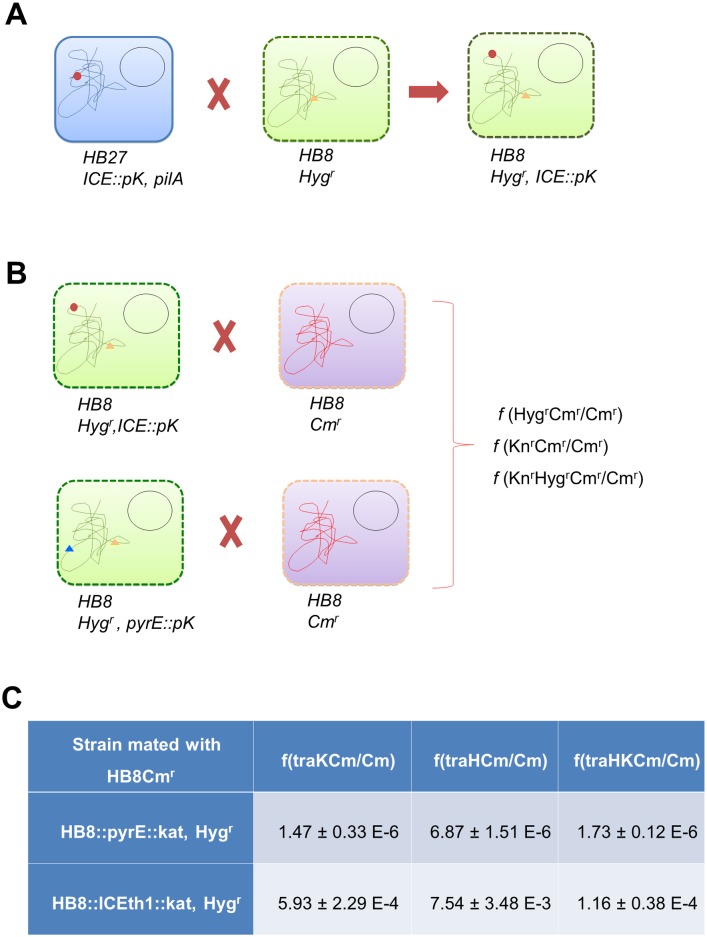
Transjugation is associated with presence of ICEth1. Scheme of the experimental design followed to unequivocally associate ICEth1 and transjugation efficiency. **(A)** ICEth1 (red dot) was labeled with Km^r^ (*ICEth1*::*pK*) in a HB27Δ*pilA4* background and transjugated into an HB8 strain labeled with Hyg^r^ (orange triangle), which naturally lacks this element. **(B)** The HB8 containing ICEth1 (Hyg^r^, Km^r^) was mated with a Cm^r^ derivative of HB8, using a Hyg^r^, *pyrE*::*pK* (blue triangle) strain as a control. **(C)** Transfer frequencies detected for the matings described in B above. Frequencies of the ICEth1-containing strain are the average value from 7 donor clones in three independent experiments. A similar number of assays were carried out for the strain lacking ICEth1.

To establish a clear relation between the presence of ICEth1 and transjugation capability in another genetic background, the experimental scheme shown in Fig 6 was followed. First, the ICEth1 was labeled with Km^r^ in a non-coding region, downstream of the *tdtA* operon in a HB27 *ΔpilA4* background to prevent this strain from serving as a recipient in transjugation. Then, mating experiments were carried out between this strain and a Hyg^r^ derivative of the HB8 strain, which does not contain the ICEth1, but does contain its *attB* site (Fig 6A). The resultant transjugants (HB8::ICEth1::*kat*, Hyg^r^) were confirmed by PCR. Finally, seven different HB8::ICEth1::*kat*, Hyg^r^ clones from the above mating were assayed in transjugation experiments with a Cm^r^ derivative of HB8 serving as recipient (Fig 6B). As a donor control, we used an HB8 strain (lacking ICEth1) with the same Hyg^r^ marker and also a Km^r^ marker, located at another gene in the chromosome (*pyrE*). Transjugation efficiencies were measured for the Km^r^ and Hyg^r^ markers with respect to the number of Cm^r^ recipients. In the absence of ICEth1, cells with a combination of resistances from both mates (Hyg or Km and Cm) were detected at frequencies ranging from 1.5–6.9 x 10^−6^ (Fig 6C), supporting that alternative horizontal gene transfer (HGT) mechanisms are acting on this strain. Such an alternative HGT mechanism has been already described, involving DNAse-resistant vesicle-protected DNA [22], and may be the most likely explanation for the observed transfer frequencies in the absence of ICEth1. In any case, presence of ICETh1 in the HB8 strain produced a 2.5 order of magnitude increase in the transfer of the Km^r^ marker (ICEth1) compared to the control strain (*pyrE*::*kat*) lacking the ICEth1. Moreover, the presence of the ICEth1 produced an even more dramatic increase (3 orders of magnitude) in the transfer efficiency of the chromosomal Hyg^r^ marker relative to the ICEth1-deficient control strain.

#### TdtA assembles into ring-shaped hexamers with ATPase activity

Sequence analysis revealed the presence of putative Walker A and B ATPase motifs in TdtA (S1 Fig). To test whether the protein actually had ATPase activity, we overexpressed and purified a His-tagged TdtA protein in *E*. *coli* (S2 Fig). Subsequently, ATPase assays were carried out at 65°C with the purified protein, revealing efficient ATP consumption that correlated with increasing TdtA concentration (S2 Fig). Presence of protein contaminants with ATPase activity or DNA from the *E*. *coli* overproducer cells was unlikely due to the harsh conditions used for purification including urea denaturation and IMAC-bound renaturation. Moreover as the ATPase assays were carried out at 65°C the putative activity of any *E*. *coli* enzyme is unlikely. As the addition of genomic dsDNA from *T*. *thermophilus* (its likely natural substrate) to these assays produced only a moderate increase in the activity (S2 Fig), the putative dependence on nucleic acids of the ATPase activity of TdtA constitutes a possibility that will require more detailed experimentation.

**Fig 7:**
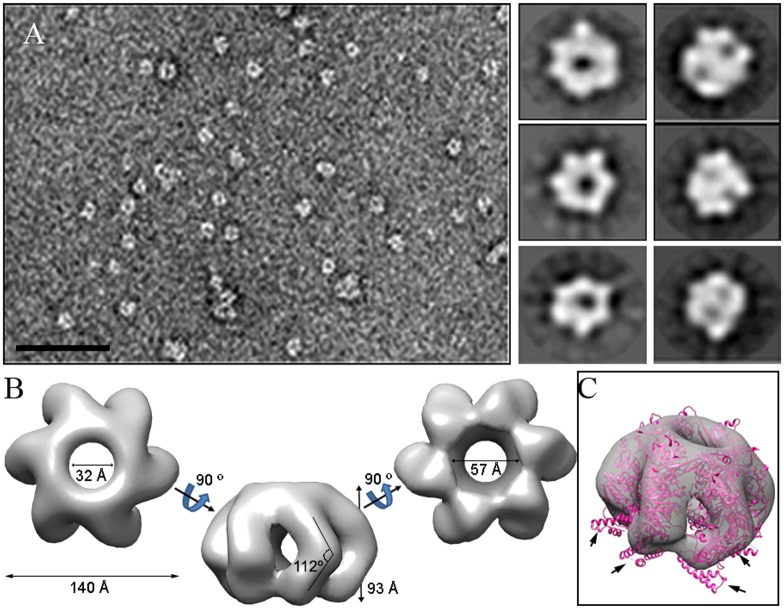
TdtA single-particle electron microscopy reconstruction. **(A)** Representative electron micrograph of a negatively stained TdtA sample; bar = 50 nm. Six two-dimensional averaged classes of the oligomeric TdtA are shown (right). **(B)** Three-dimensional reconstruction of the hexameric TdtA. **(C)** Semitransparent model of the hexameric TdtA with the fitted atomic model of the hexameric HerA from *S*. *solfataricus* (pink). Arrows indicate the HerA region (residues 216–289), which remains outside the TdtA ring.

Regular ring-shaped TdtA assemblies were detected by negative stain electron microscopy after incubation at 65°C with ATP (Fig 7A, left). In the absence of ATP or without incubation at 65°C, no such complexes were detected. Two-dimensional averaged images exhibited six-fold symmetry (Fig 7A, right), and 103,550 images were used to generate a three-dimensional reconstruction of TdtA, with a final resolution for the model estimated to be 16 Å. Oligomeric TdtA was observed to be formed by six sausage-like structures connected at their ends, with each elongated structure slightly bent at the equatorial plane (Fig 7B). The top and bottom views of this hexameric ring show a star-shaped morphology with a maximum external diameter of 140 Å and a channel with a diameter of 32–57 Å at its ends, wide enough to allow dsDNA passage.

We compared the TdtA model with structural homologs such as the hexameric HerA map from *Sulfolobus solfataricus* [23], which has similar protein monomer sizes. The HerA (Protein Data Bank (PDB) ID 4D2I) model fits quite well within the TdtA model except for a four-helix bundle (HerA residues 216–289) that remained outside (Fig 7C, arrows). These results suggest that a region of TdtA is not visible, due either to lack of contrast agent or to structural disorganization.

In conclusion, our data show TdtA to be a hexameric ATPase with an internal channel whose diameter is sufficient to accommodate dsDNA [23].

## Discussion

Cell-to-cell contact-dependent DNA transfer in *T*. *thermophilus* relies not only on the competence machinery of recipient cells to take up DNA [16], but also on a transformation-independent process for donor cells to push DNA out DNA. This *“push-pull”* model is termed “transjugation”, an intermediate process between classical conjugation and transformation. An *in silico* search for putative DNA translocases involved in DNA donation pointed to orphan members of the FtsK-HerA protein family, of which four were identified in the genome of *T*. *thermophilus* HB27. One of these (TTC0474; UniProtKB-Q72K53) was essential, consistent with a role as the actual FtsK division protein, whereas the proteins encoded by TTC0147 and TTC1430 seemed to be somehow involved in DNA repair, as corresponding knockouts were much more sensitive to UV light and/or heat shock treatments and also affecting transformation. Actually, the TTC0147 product has greater similarity to the HerA helicase of *Sulfolobus solfataricus*, described as a component of the NurA-HerA DNA-repair system [24], and also to the *Deinococcus radiodurans* HerA protein [25], which is in the same phylum as *Thermus* spp. In contrast, mutants in TTC1879 showed similar sensitivity to stress and transformation capability compared to the wild type, but a complete inability to act as a donor in transjugation experiments (Fig 1), leading us to rename its product as TdtA. In the following subsections we discuss these results.

### The role of TdtA

Mutants lacking TdtA (*push*-less) that were also defective in the transformation apparatus (*pull*-less) could not participate in transjugation with wild type strains (Fig 1), as predicted by the transjugation model [16]. Further characterization of recombinant TdtA demonstrated its ATPase activity as well as the hexameric structure common to FtsK-like DNA translocases and coupling conjugation proteins. In this context, HerA helicases involved in DNA repair have ATPase activity only in the presence of DNA [26,27]. In contrast, TdtA purified from inclusion bodies and further solubilized in urea and renatured after binding to an IMAC support (thus much likely without DNA) showed a strong *in vitro* ATPase activity in the absence of DNA, with, addition of dsDNA producing only a slight increase in the activity (S2 Fig). However, the risk of cell damage from uncontrolled ATPases such as TdtA makes more likely the existence of some class of regulation, either by timed synthesis or through posttranslational means. Considering that TdtA is present along all the culture phases some class of posttranslational control of the activity seems more likely.

The role of TdtA in transjugation could parallel that described for TraB, the hexameric ATPase required for plasmid-encoded conjugation in *Streptomyces* spp. and other Actinomycetes [9,14] or that of the TcpA protein involved in conjugal transfer of the pCW3 plasmid in *Clostridium* spp. [28]. However, whereas conjugation in *Clostridium* depends on a T4SS-like system with TcpA serving as a coupling protein, TraB functions independently, allowing for transfer of *traB*-containing plasmids as small as 10 kbp [15], and also of chromosomal genes, although with lower efficiency. Interestingly, TraB transfers dsDNA [29], in contrast to the ssDNA-transfer mediated by classical T4SS-based conjugation systems. To accommodate dsDNA, the TraB hexamer forms a 31 Å central pore [9], similar to the 32 Å central pore predicted by our electron microscopy reconstruction of the TdtA hexamer (Fig 7). Moreover, recent X-ray analysis revealed that contrary to previous reports [30], the HerA protein from *Sulfolobus solfataricus* used for the TdtA reconstruction in Fig 7 can also accommodate and push dsDNA [23]. Therefore, TdtA of *T*. *thermophilus* most likely pumps dsDNA. However, the fact that the NurA-like ssDNA-nuclease encoded upstream of *tdtA* clearly plays a role in transjugation (Fig 5) suggests that nucleolytic degradation of dsDNA is likely required in the process. As transformation in *T*. *thermophilus* is highly efficient regardless of the origin of the dsDNA [31], and transjugation depends on the competence apparatus of the recipient cell, it is likely that the actual extruded substrate through the TdtA protein is dsDNA. In this scenario, NurA could be involved either in trimming of the dsDNA substrate as proposed for the NurA protein of *Sulfolobus* spp. [23] or in repairing the targeted DNA in the chromosome of the donor.

### A model for transjugation

**Fig 8:**
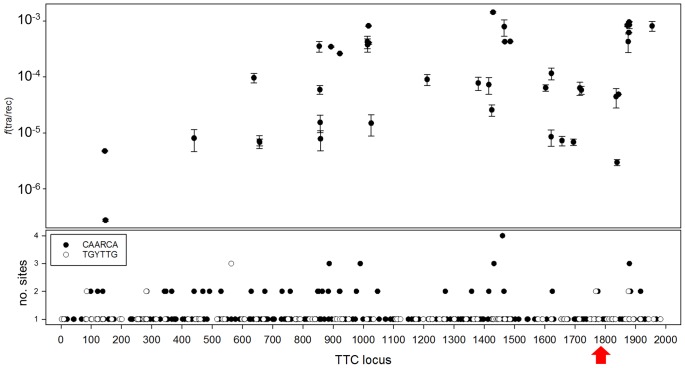
Distribution of Tth111II recognition sites and transjugation efficiencies in the HB27 chromosome. The bottom panel shows the gene locus linear map representation of the HB27 chromosome (TTC locus 1 to 1988) and the number of Tth111II recognition sites per gene found on the top (black circles) or bottom strand (empty circles). The upper panel shows the transjugation frequencies for 42 chromosomal genes (TTC::*kat* mutants described in Table 2) represented at their corresponding position in the linear map of the chromosome (n = 3). Note how the transjugation frequencies are higher in regions with greater presence of Tth111II recognition sites. The red arrow indicates the position of ICEth1.

The TraB protein of *Streptomyces* spp. recognizes 8-bp repeats through its C-terminal HTH motif [14] at the *clt*, and starts dsDNA transfer to the recipient strains. Compared to TraB, TdtA is smaller with no putative DNA binding domains detected by sequence analysis, thus the recognition of any hypothetical *oriT* would likely depend on other proteins. The best candidate for this role is the endonuclease Tth111II [21], whose deletion produced a 1,000-fold decrease in transjugation efficiency. This type IIG restriction enzyme has a rather low specific activity and preferentially nicks the top strand at position N11 downstream from its CAARCA recognition sequence. Interestingly, homologs of the same family of restriction endonucleases are also found in the *tdtA*-like gene clusters of *T*. *scotoductus* DSM8553 (WP_020700619.1, 50% identity), and *Chloroacidobacterium thermophilus* B (WP_014099240.1, 66% identity). Thus, it is tempting to speculate about the role of these restriction enzymes in the nicking of DNA to signal the origin of transfer sites during transjugation in *Thermus spp* or in conjugation-like processes in other bacteria. In this context, it is of note the existence of at least a conjugative relaxase described that contains a C-terminal domain with structural similarity to restriction endonucleases [32], suggesting some connection of these two classes of enzymes. As the transjugation process in *T*. *thermophilus* seems to start simultaneously at several points in the chromosome [16], and having in mind the ancestral character of the genus *Thermus* spp, it is tempting to speculate on primitive restriction-like enzymes acting in HGT processes. Actually, a map of transfer frequencies for 42 genes against their chromosome locations shows greater transfer of genes located in regions with more Tth111II recognition sites, whereas less-frequently transferred genes are found in regions with fewer recognition sites (Fig 8). Therefore, a tentative but preliminary hypothesis involves Tth111II as the enzyme that recognizes multiple origin of transfer for the concerted action of a likely NurA-TdtA complex.

### TdtA is encoded by a new class of ICE

In a bioinformatics study searching for mobility of R-M systems, Furuta and co-workers identified the TTC1877 (encoding Tth111II) and TTC1880 (encoding a putative methylase) genes of *T*. *thermophilus* HB27 as part of an integrated element that was absent in the HB8 strain [33]. Our data support that the 14,857 bp region located between 47-bp direct repeats (*i*.*e*., *attL* and *attR*) actually constitutes the first active ICE described for *T*. *thermophilus* (ICEth1). However, our studies on ICEth1 excision by PCR and frequency of loss during exponential growth (up to 8%) suggested that the ICEth1 does not replicate after excision from the chromosome like other ICEs do [34]. Therefore, for the ICEth1 to be maintained in the population, a continuous mobilization and invasion process must take place in order to spread to those cells that had lost the element during exponential growth.

The ability of ICEth1 to spread to ICE-less strains was shown using the HB8 as recipient. In our experiments, the new ICEth1^+^ HB8 behaves as a donor of the inherited ICEth1, as expected for any ICE. However, in contrast to most ICEs, the ICEth1 promotes the efficient transfer of genes from other locations in the genome, in what seems to be a sort of generalized transfer that mimics the well-known generalized transduction in classical bacterial HGT processes. Thus, in the absence of selectable properties encoded by ICEth1, it is tempting to speculate that the selection of favorable phenotypic traits (*i*.*e*., anaerobic respiration, heavy metal resistance, UV resistance, etc.) under given environmental conditions would also favor the co-selection and spreading of ICEth1 itself throughout a population of recipient strains. In this context, the noteworthy ability of ICEth1-mediated transjugation to escape the Argonaute surveillance system, which is an effective barrier against environmental [16] and vesicle protected DNA [22], enhances its relevance in intraspecies HGT and facilitates its own spreading.

## Methods

### Bacterial strains and growth conditions

Table 2 summarizes the bacterial strains employed in this study. *Escherichia coli* strains DH5α (for plasmid construction) and BL21 (DE3) (for protein overexpression and purification) were grown at 37°C in LB (Luria-Bertani) selective media. Aerobic growth of *T*. *thermophilus* strains was carried out with rotational shaking (150 rpm) at 60°C in TB (*Thermus* broth) liquid or solid media, unless otherwise indicated. Selection with Km (30 μg/ml), ampicillin (Am, 100 μg/ml), Cm (20 μg/ml) and/or Hyg (100 μg/ml) was employed as required.

**Table 2:** Bacterial strains used in this work.

Strain	Genotype	Phenotype/use	Source
*E*. *coli* DH5α	*supE44 ΔlacU169 (Φ80 lacZΔM15) hsdR17*, *recA1*, *endA1*, *gyrA96*, *thi-1 relA1*	Ordinary cloning	[35]
*E*. *coli* BL21 (DE3)	*F*^-^ *ompT gal dcm lon* HsdSB (r_B_^-^m_B_^-^) λ(DE3 [*lacI lacUV5-T7* gene 1 *ind1 sam7 nin5*])	Protein overexpression and purification	[36]
***T*. *thermophilus strains***			
HB27wt	*ATCC BAA-163/DSM7039*	Wt	Y. Koyama
NAR1	*[pTT27*::*nar]*	wt. NCE, partial denitrifer	[37]
HB8	*ATCC 27634*	ICEth1-less	Y. Koyama
HB27^EC^	*HB27 ago*::*agoISTth7*	enhanced competence	This work
Wt^Cm^		HB27 Cm^r^	This work
*Δago*	*HB27 Δago*	Argonaute less	[38]
*ΔpilA4*	HB27^EC^ *ΔpilA4*	Non competent	[16]
*ΔpilA4*::*hyg*	HB27^EC^ *ΔTTC0858*::*hyg*	Non competent	[16]
*ΔtdtA*	HB27^EC^ *ΔTTC1879*	Deficient in DNA donation	This work
*tdtA*::pk	HB27^EC^*TTC1879*::*pK18*	Km^r^ Deficient in DNA donation.	This work
*ΔtdtA*::*kat*	HB27^EC^ *ΔTTC1879*::*kat*	Km^r^. Deficient in DNA donation.	This work
*ΔtdtA*::*hyg*	HB27^EC^ *ΔTTC1879*::*hyg*,	Hyg^r^. Impaired in DNA donation.	This work
*tdtA*::*kat pilA4*	HB27^EC^ *ΔTTC1879*::*kat*, *ΔpilA4*	Km^r^. Deficient in DNA donation and transformation.	This work
Wt^H^	HB27^EC^*ΔTTC0313*::*hyg*	Hyg^r^	[16]
Nar *tdtA*::*kat*	NAR1 [*ΔTTC1879*::*kat*]	Nitrate respiring. Km^r^	This work
Nar *TTC*::*hyg*	NAR1 [*ΔTTC0313*::*hyg*]	Nitrate respiring. Hyg^r^	[16]
*gdh*::*kat*	HB27 [*ΔTTC1211*::*kat*]	Km^r^	[37]
HB8 *hyg*	HB8 [*ΔTTC0313*::*hyg*]	ICEth1-less. Hyg^r^	This work
HB8 *Cm*^*r*^	HB8 spontaneous chloramphenicol resistant	ICEth1-less. Cm^r^	This work
HB8::ICEth1::*kat*, Hyg^r^	HB8 [*ΔTTC0313*::*hyg*], ICEth1::pK18	ICEth1, Km^r^, Hyg^r^	This work
*tdtA*YFPph	HB27^EC^ [*TTC1879-sYFP*:*pH118]*	Hyg^r^. Single copy expression of TdtA fused to sYFP	This work
*ΔtdtA*, *ΔpilA4* PMH	HB27^EC^ *ΔTTC1879*::*kat*, *ΔpilA4* [pMH::*TTC1879*]	Complementation of TdtA	This work
*ΔtdtA*, *ΔpilA4* PMHY	HB27^EC^ *ΔTTC1879*::*kat*, *ΔpilA4* [pMH::*TTC1879*::*sYFP*]	Complementation of TdtA with TdtA-sYFP	This work
ICEth13’kat	HB27 TTC1880-TTC1881::pK18	Intergenic tag (TTC1880-TTC1881) of kanamycin marker.	This work
*ΔTh111II*::*kat*, *ΔpilA4*	HB27^EC^ *ΔTTC1877*::*kat*	Km^r^. Impaired in DNA donation and transformation	This work
Δ*nurA*::*kat*, Δ*pilA4*	HB27^EC^ Δ*TTC1878*::*kat*	Km^r^. Impaired in DNA donation and transformation	This work
Δ*TTC0147*::*kat*	HB27^EC^ Δ*TTC0147*::*kat*	Km^r^. Affected in stress survival	This work
Δ*TTC1430*::*kat*	HB27^EC^ Δ*TTC1430*::*kat*	Km^r^. Affected in stress survival	This work
Δ*TTC1844*::*kat*	HB27^EC^ Δ*TTC1844*::*kat*	Km^r^. Affected in stress survival and transformation	[16]
Δ*gdh*::*kat*	HB27 Δ*TTC1211*::*kat*	Km^r^	[16]
Δ*pilA4*::*kat*	HB27 Δ*TTC0858*::*kat*	Km^r^. Impaired in transformation	[16]
*TTC1017*::*kat*	HB27 Δ*TTC1017*::*kat*	Km^r^. Impaired in transformation	[16]
*TTC1622*:*kat*	HB27 Δ*TTC1622*::*kat*	Km^r^. Impaired in transformation	[39]
*TTC854*::*kat*	HB27 Δ*TTC0854*::*kat*	Km^r^	[40]
*TTC1621*::*kat*	HB27 Δ*TTC1621*::*kat*	Km^r^	[39]
*TTC857*::*kat*	HB27 Δ*TTC0857*::*kat*	Km^r^	[40]
*TTC0856*::*kat*	HB27 Δ*TTC0856*::*kat*	Km^r^	[40]
*TTC1716*::*kat*	HB27 Δ*TTC1716*::*kat*	Km^r^	[40]
*TTTC1603*::*kat*	HB27 Δ*TTC1603*::*kat*	Km^r^	[39]
*TTC1415*::*kat*	HB27 Δ*TTC1415*::*kat*	Km^r^	[16]
*TTC0638*::*kat*	HB27 Δ*TTC0638*::*kat*	Km^r^	[16]
*TTC0893*::*kat*	HB27 Δ*TTC0893*::*kat*	Km^r^	[16]
*TTC1425*::*kat*	HB27 Δ*TTC1425*::*kat*	Km^r^	This work
*TTC1429*::*kat*	HB27 Δ*TTC1429*::*kat*	Km^r^	This work
*TTC0474*::*kat*	HB27 Δ*TTC0474*::*kat*	Km^r^	This work
*TTC0656*::*kat*	HB27 Δ*TTC0656*::*kat*	Km^r^	This work
*TTC1026*::*kat*	HB27 Δ*TTC1026*::*kat*	Km^r^	This work
*TTC1878*::*kat*	HB27 Δ*TTC1878*::*kat*	Km^r^	This work
*TTC1877*::*kat*	HB27 Δ*TTC1877*::*kat*	Km^r^	This work
*TTC1380*::*kat*	HB27 Δ*TTC1380*::*kat*	Km^r^	This work
*TTC1013*::*kat*	HB27 Δ*TTC1013*::*kat*	Km^r^	[40]
*TTC1873*::*kat*	HB27 Δ*TTC1873*::*kat*	Km^r^	This work
*TTC440*::*kat*	HB27 Δ*TTC0440*::*kat*	Km^r^	[40]
*TTC1695*::*kat*	HB27 Δ*TTC1695*::*kat*	Km^r^	This work
*TTC1468*::*kat*	HB27 Δ*TTC1468*::*kat*	Km^r^	This work
*TTC1839*::*kat*	HB27 Δ*TTC1839*::*kat*	Km^r^	This work
*TTC1836*::*kat*	HB27 Δ*TTC1836*::*kat*	Km^r^	This work
*TTC1876*::*kat*	HB27 Δ*TTC1876*::*kat*	Km^r^	This work
*TTC1466*::*kat*	HB27 Δ*TTC1466*::*kat*	Km^r^	This work
*TTC922*::*kat*	HB27 Δ*TTC0922*::*kat*	Km^r^	[16]
*TTC1955*::*kat*	HB27 Δ*TTC1955*::*kat*	Km^r^	This work
*TTC1721*::*kat*	HB27 Δ*TTC1721*::*kat*	Km^r^	This work
*TTC1486*::*kat*	HB27 Δ*TTC1486*::*kat*	Km^r^	This work
*TTC1657*::*kat*	HB27 Δ*TTC1657*::*kat*	Km^r^	This work
*TTC657*::*kat*	HB27 Δ*TTC0657*::*kat*	Km^r^	This work
*TTC1880*::*kat*	HB27 Δ*TTC1880*::*kat*	Km^r^	This work
*TTC145*::*kat*	HB27 Δ*TTC0145*::*kat*	Km^r^	This work
*TTC1014*::*kat*	HB27 Δ*TTC1014*::*kat*	Km^r^	[40]
*TTC1018*::*kat*	HB27 Δ*TTC1018*::*kat*	Km^r^	[40]
*TTC1844*::*kat*	HB27 Δ*TTC1844*::*kat*	Km^r^	[16]

### Generation of bacterial mutants

The plasmids employed in this work are listed in S1 Table. For initial screening of gene functionality, single-insertion mutants were obtained by recombination with central regions of the target gene cloned in suicide vectors pK18 or pH118, encoding thermostable resistance to Km or Hyg, respectively. For further genetic analysis, deletion derivatives were generated by double recombination between specific upstream and downstream target arms separated by the *kat* or the *hyg* genes (encoding resistance to Km and Hyg, respectively). For marker-less mutants, these *Δgene*::*cassette* mutants were transformed again with a similar upstream-downstream construct and cloned into a suicide vector carrying a different thermostable resistance gene. After isolation of single recombinants with both antibiotic resistances, spontaneous loss of both markers by back recombination allowed the isolation of marker-less *Δgene* mutants. All mutants were confirmed by PCR and sequencing. Spontaneous Cm^r^ strains were obtained when necessary. The isolation of mutants in non-competent *ΔpilA4* cells required electroporation.

Isolation of strains expressing fusions to thermostable fluorescent proteins was performed by fusing either the whole gene or its C-terminal region to superfolder yellow fluorescent protein (sYFP, [41]). Fusions of C-terminal regions to sYFP were cloned into suicide plasmid pK18, and subsequently transformed into *T*. *thermophilus*. Transformants were checked by fluorescence microscopy, PCR and sequencing.

Complementation of mutants was carried out using derivatives of plasmid pMH184 (Hyg^r^), which constitutively expressed the corresponding gene at moderate levels.

### Transformation and transjugation assays

Quantitative transformation assays were performed as described [16,42] using exponential cultures grown at 65°C and 10 to 200 ng of genomic or plasmid DNA. Transformation frequencies were estimated by the number of CFU on selective plates per viable cell.

Mating experiments were conducted as described by Blesa *et al* [16]. Briefly, 100 μl of saturated cultures of Hyg^r^ and Km^r^ strains were mixed in the presence of DNase I (5 units; Roche), washed in 1 volume of TB, resuspended in 10 μl of TB containing DNase I (5 units; Roche) and plated onto sterile 0.22 μm nitrocellulose filters (GSWP, Millipore) on TB agar plates prior to incubation for 4 h at 60°C. Cells on the filters were then resuspended in TB and appropriate serial dilutions were plated onto selective agar plates. Transjugation frequencies were expressed as the number of CFU grown in selective media (Hyg plus Km, and in some experiments, Cm) per CFU grown on agar plates with Hyg or Cm.

Statistical analysis of the transfer frequencies was performed using SPSS ^®^Statistics v.21.0 (SPSS Inc., Chicago, USA; 2008), considered statistically significant when *p*–value < 0.05. Inferential and comparative assays were performed when necessary and included Student’s t-tests, Kruskal-Wallis one-way analysis, Wilcoxon tests and one-way ANOVA tests.

The parenthood analysis of transjugants in Fig 2 was carried out by collecting in a single pool all the colonies grown on the selection plates of the assays described above (>10^3^ transjugants). Aliquots of these pools were used for genomic DNA isolation and for membrane protein profile analysis. PCR assays specific for the NAR1 and HB27 strains were carried out using primer sets nrcE_fw/nrcE_rev and TTP0220_fw/TTP0220_rev, respectively (S2 Table). Membrane isolation was done after breaking the cells by sonication (4°C, 3 pulses of 1 min with 0.5 s cycles, 0.9 intensity, Labsonic, Braun), elimination of unbroken cells by low speed centrifugation (5,000 x *g* 5 min) and further recovering of membrane insoluble fraction by higher speed centrifugation (15,000 x *g* for 20 min). Membrane proteins were analyzed by PAGE and gene staining as described below.

### Stress assays

Resistance to UV radiation and heat shock were measured as the ratio of viable cells after 60 min of UV light exposure (Osram Sylvania G8T5, λ = 254 nm) and 60 min incubation at 90°C, and expressed as the ratio with respect to untreated controls. The strain used as wild type in these analyses was the *Δgdh*::*kat* mutant, to control for any role of Km resistance in the assays. Statistical significance of differences between mutants and wild type both for UV and “heat shock” assays was checked as above.

### Recombinant protein expression and purification

The *tdtA* gene was cloned into pET28-b and electroporated into BL21(DE3) *E*. *coli* cells. Cells were grown in LB with Km at 30°C to an OD_600_ = 0.6, at which point 0.4 mM IPTG was added and incubated for 12 h at 24°C. Cells were harvested by centrifugation (23,700 x g for 20 min at 4°C) and 0.4 g of wet weight cells were resuspended in 30 ml of buffer A (25 mM sodium phosphate buffer pH 8, 100 mM NaCl and 0.1% Tween 20). Lysis was accomplished by sonication (3 pulses for 1 min, 0.8 power, 0.9 intensity, 0.5 s of cycle frequency, in a Labsonic U, B. Braun device), and cell debris was removed by centrifugation (23,700 x *g* for 20 min at 4°C). The insoluble fraction, containing inclusion bodies of the His-tagged TdtA protein, was washed in 7 ml of buffer A and further incubated for 30 min at 30°C in 4 ml of the same buffer with Triton 0.2% (w/v). After detergent removal by centrifugation (23,700 x g for 15 min at 4°C) the pellet was washed again with 1.5 ml buffer A and centrifuged as before. In order to solubilize aggregated proteins, the pellet was resuspended in buffer A containing 6 M urea (Merck) and incubated for 12 h at 4°C. After centrifugation to remove contaminants, the supernatant was loaded onto a pre-equilibrated Cobalt TALON resin (ClonTech Laboratories, Inc.). Renaturation by step-down urea gradient was performed and the final 2 M urea fraction eluted from the column with imidazole was dialyzed against 25 mM phosphate buffer pH 8.0 with 100 mM NaCl and loaded again onto pre-equilibrated Cobalt TALON resin. Proteins were eluted with 200 mM imidazole, dialyzed against the above buffer and stored at -20 and 4°C with 50% and 10% glycerol, respectively. Glycerol-free purified protein fractions were used directly in electron microscopy and ATPase assays.

### Single-particle electron microscopy and image processing

Samples of purified TdtA (2–5 μl at 1.5 mg/ml in 25 mM Tris- HCl pH 7.5), previously incubated with 1–10 mM ATP (30 min at 65°C with shaking), were applied to glow-discharged carbon-coated grids for 2 min. Grids were washed twice with water and negatively stained with 2% (w/v) aqueous uranyl acetate. Electron microscopy images were recorded with a CCD camera (4k x 4k TEMCam-F416, TVIPS) on a JEOL 1010 JEM electron microscope operating at 80 kV. Images were recorded at a sampling rate of 2.44 Å/pixel, with an under focus ranging from 0.7 to 1.5 μm.

General image processing operations were performed using Xmipp software [43], and graphics were produced by UCSF Chimera [44]. The contrast transfer function (CTF) was corrected with Ctffind3 [45] and images were down-sampled to a factor of 2 (final sampling ratio 4.88 Å/pixel). The Xmipp automatic picking routine was used to select 103,745 TdtA particles which were classified using a reference-free clustering approach with the CL2D program [46] to select a homogeneous population of 103,550 particles. An artificial noise model was used as a starting reference for iterative angular refinement using the EMAN program [47]. The resulting model was selected and refined using the Xmipp iterative projection matching routine [48]. After independent refinement processes, 90% of particles were included in the final three-dimensional reconstruction and the resolution of the model was determined by the Fourier shell correlation (FSC) criterion between independent half-dataset maps applying a correlation limit of 0.5.

The CHIMERA fitting routine was used to dock the HerA atomic model in the three-dimensional map of TdtA after initial manual placement. Each protomer of the crystallographic hexamer was fitted in the cryo-EM map as an independent rigid body.

### Protein detection

For cell fractionation, cell samples were recovered and washed by centrifugation (5000 x *g* 5 min), resuspended in 50 mM phosphate buffer, pH 7, and broken by sonication in the same buffer (4°C, 3 pulses of 1 min with 0.5 s cycles, 0.9 intensity, Labsonic, Braun). Residual unbroken cells and large cell fragments were eliminated by centrifugation (5,000 x *g* 5 min) and the soluble fractions was obtained after two consecutive 15,000 x *g* for 20 min centrifugation steps to avoid contamination with membranes. The insoluble fraction was also washed twice by centrifugation in the same buffer and conditions before SDS-PAGE and western blot analysis. PAGE protein analysis was carried out by standard procedures in 12% acrylamide/bis-acrylamide gels stained with Coomassie-Blue. Inmunodetection of TdtA and HepA proteins in the soluble and insoluble cell fractions of *T*. *thermophilus* HB27 was carried out by western blot using rabbit antiserum raised against the HepA protein from *T*. *thermophilus*, which also cross-reacts with its TdtA homolog, allowing the detection of both proteins simultaneously. Inmunodetection of protein fusions to sYFP was carried out with commercial antiserum. Goat anti-rabbit secondary antibodies conjugated to horseradish peroxidase were used for specific chemiluminescent detection of the protein (ECL, Amersham International).

### Measurement of ATPase activity

ATP hydrolysis was estimated using the luciferin-luciferase ATP Bioluminescence Assay Kit CLS II (Roche), following manufacturer’s instructions. Different amounts of the analyzed protein were incubated for 1 h at 65°C in a buffer containing 5 mM MgSO_4_, 50 mM NaCl, 25 mM Tris-HCl pH 7.5 and 0.1 mM of ATP (Sigma-Aldrich). End-point kinetic ATPase assays were run in triplicate and results were expressed as percentage of ATP consumed against protein concentration (nM). No-protein controls, both with and without substrate, were included in every assay.

### Reverse transcription PCR

RNA was isolated from *T*. *thermophilus* HB27 using an RNeasy Mini Kit (QIAGEN), and after DNAse I treatment (RQ1, Promega), all samples were tested by conventional PCR to verify absence of DNA contamination. Reverse transcription was performed using the SuperScript III first strand synthesis kit (Invitrogen) according to the manufacturer’s instructions. Subsequent PCRs to amplify cDNA were performed using *Pfu* Ultra II Fusion HS DNA Polymerase (Agilent Technologies) with the primers indicated in S2 Table.

## Supporting information

S1 Fig Multiple sequence alignments of TdtA (TTC1879). BLASTp results for TdtA with its best homologs ordered by highest TdtA sequence similarity: *Chloracidobac* (*Chloracidobacterium thermophilum*), *T*. *scotoductus (Thermus scotoductus SA01)*, *T*. *antranikianii* (*Thermus antranikianii*) and *Thermotoga (Thermotoga napholitana)*. Common amino acids among the five sequences are represented in red, with conserved ATPase (Walker A, Walker B) and HAS-barrel motifs shown within red boxes.
(TIF)

S2 Fig Purification and ATPase activity of TdtA. SDS-PAGE gels showing: **(A)** total protein content of *E*. *coli* BL21 cells carrying plasmid pAB201 before (ni) and after induction (i). **(B)** proteins of the soluble (s) and insoluble (p) cell fractions. **(C)** IMAC affinity-purified TdtA protein (pp). Lane M corresponds to protein size markers of: 97.4, 66.2, 45, 31, 21.5 and 14.4 kDa. **(D)** % of ATP consumed after incubation for 1 h at 65°C with the indicated concentrations of TdtA in the absence of DNA (diamonds) or in the presence of 1 mg (1.2 nM) of genomic dsDNA from *T*. *thermophilus* (squares). Initial concentration of ATP was 10^−4^ M.
(TIF)

S1 Table Plasmids employed in this work. (DOCX)

S2 Table Oligonucleotides used in this work. (DOCX)
