# Supplementary figures and images for: The transjugation machinery of Thermus thermophilus: Identification of TdtA, an ATPase involved in DNA donation
Source: PLoS Genet. 2017 Mar 10;13(3):e1006669. doi: 10.1371/journal.pgen.1006669 (PMC5365140; doi:10.1371/journal.pgen.1006669)

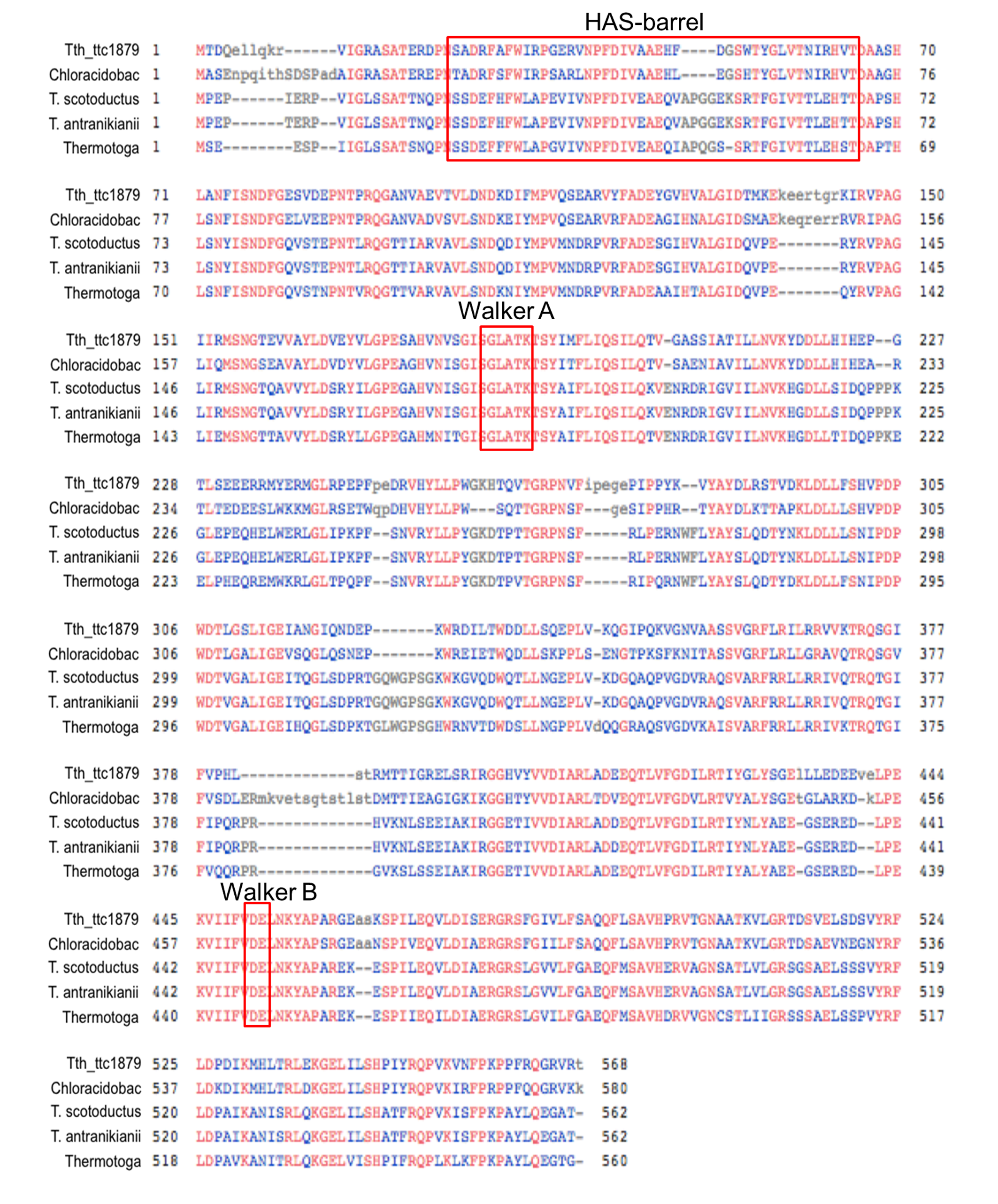

Supplement: S1 Fig — BLASTp results for TdtA with its best homologs ordered by highest TdtA sequence similarity: Chloracidobac (Chloracidobacterium thermophilum), T. scotoductus (Thermus scotoductus SA01), T. antranikianii (Thermus antranikianii) and Thermotoga (Thermotoga napholitana). Common amino acids among the five sequences are represented in red, with conserved ATPase (Walker A, Walker B) and HAS-barrel motifs shown within red boxes. (TIF) [file pgen.1006669.s001.tif]

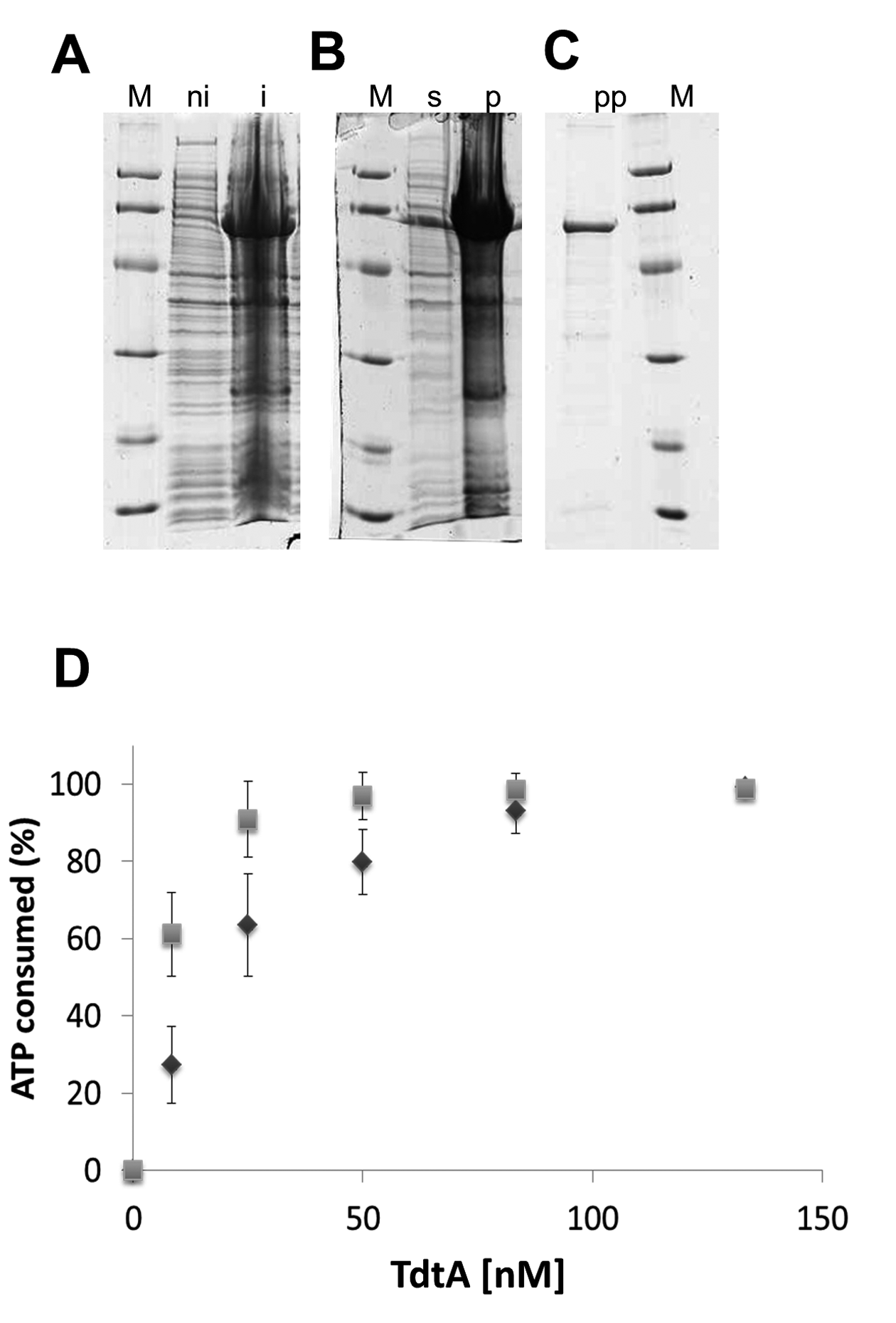

Supplement: S2 Fig — SDS-PAGE gels showing: (A) total protein content of E. coli BL21 cells carrying plasmid pAB201 before (ni) and after induction (i). (B) proteins of the soluble (s) and insoluble (p) cell fractions. (C) IMAC affinity-purified TdtA protein (pp). Lane M corresponds to protein size markers of: 97.4, 66.2, 45, 31, 21.5 and 14.4 kDa. (D) % of ATP consumed after incubation for 1 h at 65°C with the indicated concentrations of TdtA in the absence of DNA (diamonds) or in the presence of 1 mg (1.2 nM) of genomic dsDNA from T. thermophilus (squares). Initial concentration of ATP was 10−4 M. (TIF) [file pgen.1006669.s002.tif]
