# Supplementary material for: The transjugation machinery of Thermus thermophilus: Identification of TdtA, an ATPase involved in DNA donation
Source: PLoS Genet. 2017 Mar 10;13(3):e1006669. doi: 10.1371/journal.pgen.1006669 (PMC5365140; doi:10.1371/journal.pgen.1006669)
Supplement: S1 Table — (DOCX) [file pgen.1006669.s003.docx]

**S1 Table. Plasmids employed in this work**

| **Plasmid** | **Description/Use** | **Reference** |
| --- | --- | --- |
| pET28b(+) | Km^r^, *lacI*, *E. coli* expression gene dependent on Ö10 promotor from RNA polymerase of T7 phage. Includes fusion of a 6xHis-tag at N-terminal site. Protein overexpression for purification | Novagen |
| pUC19/18 | Am^r^, P-lac-lacZ'. Cloning | [1] |
| pUC19*::kat* | Am^r^, Km^r^. pUC19 derivative with Km resistance cassette cloned at *XbaI* site. Cloning | This work |
| pUC19*::hyg* | Am^r^, Hyg^r^. pUC19 derivative with Hyg resistance cassette inserted at *XbaI* site. Cloning | This work |
| pK18 (pK118) | Km^r^. Suicide vector for mutagenesis. Cloning, generation of single insertion mutants | [2] |
| pH118 | Hyg^r^. Suicide vector for mutagenesis. Cloning, generation of single insertion mutants | Laboratory plasmid |
| pMK184 | Km^r^. Cloning in *T. thermophilus* | Laboratory plasmid |
| pMH184 | Hyg^r^. Cloning in *T. thermophilus* | Laboratory plasmid |
| pWUR | Bleo^r^. Cloning. Transformation and expression in *T. thermophilus* | [3] |
| pUC19::TTC0313::hyg | Hyg^r^, Am^r^. pUC19 derivative harboring *TTC0313* gene (*XbaI/EcoRI*), interrupted by *hph5* resistance gene cassette | [4] |
| pMHPnqosYFP | Hyg^r^. pMH184 derivative harboring the reporter superfolder YFP (sYFP), controlled by the promoter Pnqo (*XbaI/HindIII*).*T. thermophilus* replicative, Protein fusion mutants | [2] |
| pET11a::TTHA0522 | Am^r^. Expression vector encoding HB8 gene *TTHA0522*, tailed with a His-tag at N-terminus (*BglII/EcoRI*) | RIKEN BioRseource Center |
| pAB22 | pUC19::*ΔpilA4:kat*. Km^r^. *pilA4* deletion, replaced by the *kat* cassette, flanked by upstream and downstream sequences of *pilA4* gene (*EcoRI/HindIII*) | [5] |
| pAB52 | puC19:: *ΔpilA4::hyg*. Am^r^, Hyg^r^. pUC19 derivative, enclosing up- and downstream flanking regions of *pilA4* gene, which is replaced by the *hph5* cassette (*XbaI*) | [5] |
| pAB54 | puC19::*ΔpulE::kat*. Am^r^, Km^r^. pUC19 derivative, enclosing up- and downstream flanking regions of *pulE* gene (TTC1844), which is replaced by the *kat* cassette (*XbaI*) | This work |
| pAB70 | pk118::*TTC1879.* Km^r^. Insertion of the *kat* cassette interrupting *TTC1879* gene (*tdtA*) (*HindIII/BamHI*) | This work |
| pAB71 | pH118:*:TTC1879*. Hyg^r^. Insertion of the *hph5* cassette interrupting *TTC1879* gene (*tdtA*) (*HindIII/BamHI*) | This work |
| pAB106 | pUC19*::∆TTC1879::kat*. Am^r^, Km^r^. pUC19 derivative where *tdtA* gene is replaced by the *kat* cassette, flanked by upstream and downstream regions of *TTC1879* (*tdtA*) (*HindIII/BamHI*) | This work |
| pAB107 | pUC19:: *∆TTC1879::hyg*. Am^r^, Hyg^r^. pUC19 derivative where *tdtA* gene is replaced by the *hph5* cassette, flanked by upstream and downstream regions of *TTC1879* (*tdtA*) (*HindIII/BamHI*) | This work |
| pAB109 | pMH::c1879. Hyg^r^. Complementation. pMH184 derivative expressing *tdtA* gene (*BamHI/HindIII*) | This work |
| pAB110 | pMK::c1879. Km^r^. Complementation. pMK184 derivative expressing *tdtA* gene (*BamHI/HindIII*) | This work |
| pAB122 | pUC18*::cptB::kat*. Am^r^, Km^r^. pUC18 derivative where *TTC1430* gene is replaced by the *kat* cassette, flanked by upstream and downstream regions of *TTC1430* (*HindIII/EcoRI*) | This work |
| pAB181 | pK18::*TTC0474*. Km^r^. Insertion of the *kat* cassette interrupting *TTC0474* gene (*ftsK*) (*EcoRI/HindIII*) | This work |
| pAB201 | pET28b::*TTC1879.* Km^r^. Expression of *T. thermophilus* HB27 *TTC1879* gene (*tdtA*) (*NdeI//HindIII*) | This work |
| pAB207 | pUC18::*ΔTTC0147::kat*. Am^r^, Km^r^. pUC18 derivative, enclosing up- and downstream flanking regions of *hepA* gene (*TTC0147*), which is replaced by the *kat* cassette (*XbaI*) | This work |
| pAB213 | pH:*TTC1879*::sYFP. Hyg^r^. Recombinant plasmid, pH118 derivative where the C-terminus of TdtA (*TTC1879*) is fused to sYFP (*BcuI*) | This work |
| pAB224 | pMH::*TTC1879*::sYFP. Hyg^r^. Expression plasmid, pMHPnqosYFP derivative where TdtA (*TTC1879*) is fused to sYFP (*BcuI*) | This work |
| pAB283 | pK18::*ICEth1*.Km^r^. Insertion of the *kat* cassette in the intergenic region downstream *TTC1880*. Mobility of ICEth1 (*HindIII/XbaI*) | This work |
| pAB298 | pUC18::*ΔTTC1877::kat*. Am^r^, Km^r^. pUC18 derivative, enclosing up- and downstream flanking regions of the restrictase *tth111II* gene (*TTC1877*), which is replaced by the *kat* cassette (*XbaI*) | This work |
| pAB153 | pK18::*pyrE*. Km^r^. Insertion of the *kat* cassette in the *pyrE* gene (*TTC1380*). (*HindIII/EcoRI*) | This work |
| pIB008 | pUC18::*ΔTTC1878::kat*. Am^r^, Km^r^. pUC18 derivative, enclosing up- and downstream flanking regions of *nurA* gene (*TTC1878*), which is replaced by the *kat* cassette (*XbaI*) | This work |

**References to S3 Table**

1. Vieira J, Messing J (1982) The pUC plasmids, an M13mp7-derived system for insertion mutagenesis and sequencing with synthetic universal primers. Gene 19: 259-268.

2. Cava F, Zafra O, Magalon A, Blasco F, Berenguer J (2004) A new type of NADH dehydrogenase specific for nitrate respiration in the extreme thermophile *Thermus thermophilus*. Journal of Biological Chemistry 279: 45369-45378.

3. Brouns SJJ, Wu H, Akerboom J, Turnbull AP, de Vos WM, et al. (2005) Engineering a Selectable Marker for Hyperthermophiles. Journal of Biological Chemistry 280: 11422-11431.

4. Zafra O, Ramirez S, Castan P, Moreno R, Cava F, et al. (2002) A cytochrome *c* encoded by the *nar* operon is required for the synthesis of active respiratory nitrate reductase in *Thermus thermophilus*. FEBS Lett 523: 99-102.

5. Blesa A, César CE, Averhoff B, Berenguer J (2014) Non canonical cell-to-cell DNA transfer in *Thermus* spp. is insensitive to Argonaute-mediated interference. Journal of Bacteriology.
