# Supplementary material for: The transjugation machinery of Thermus thermophilus: Identification of TdtA, an ATPase involved in DNA donation
Source: PLoS Genet. 2017 Mar 10;13(3):e1006669. doi: 10.1371/journal.pgen.1006669 (PMC5365140; doi:10.1371/journal.pgen.1006669)
Supplement: S2 Table — (DOCX) [file pgen.1006669.s004.docx]

**S2 Table. Oligonucleotides used in this work.** In capital letters, the annealing sequence and underlined, the cloning restriction sites employed.

| **Primer (Use)** | **Sequence 5' -> 3'** |
| --- | --- |
| 1Fw (RT-PCR) | GCCACACGGTAAGCTCC |
| 2Rv (RT-PCR) | GAACACCCAACTTCTGAGCC |
| 3Fw (RT-PCR) | GCATTCCCAGATATGAACCG |
| 4Rv (RT-PCR) | AACCGTCCAGGAAATAGCG |
| 5Fw (RT-PCR) | GGGTGTCGTCAAAGTGGAA |
| 6Rv (RT-PCR) | GTCCGTCACATGGCGAAT |
| 7Fw (RT-PCR) | ACGGGCAATGCTGCTAC |
| 8Rv (RT-PCR) | GCTGAAGCGCACGTAGAAC |
| 9Fw (RT-PCR) | GGGAGCGATATGTGCCTTT |
| 10Rv (RT-PCR) | TTCTGGTGTACGCCCTGG |
| 11Rv (ICEth1 excision) | GGTGCTGGACCTGGAAGAAC |
| 12Fw (ICEth1 excision) | CGTCACCCTGAAGGAGACC |
| 13Fw (ICEth1 excision) | CTTGGTCGCCCGAGAGTCAAG |
| 14Rv (ICEth1 excision) | GAGCTTCCGATGGCGTC |
| TTC0313dir | CTTTACGAGGCCCTCTTGGAG |
| TTC0313rev | CCACCGCTCGGGGAC |
| AB92 (check *pilA4* deletion) | aaaTGCTGAAGCTTGGCGGCAAC |
| AB93 (check *pilA4* deletion) | aaaAGAATTCGGGAGTTAGGCTTGGGATTGTG |
| AB169 (insertion *tdtA*) | atcaagcttGAGTTATTGGCCGCGCTTC |
| AB170 (insertion *tdtA*) | aaaccatggCATGCGGGTGCTCAGGTG |
| AB175 (deletion *tdtA*) | aaatctagaGACGGCCTCCAGATAGCCATC |
| AB176 (deletion *tdtA*) | atcaagcttCATAGACGAGCAGGTGCTCCAG |
| AB177 (deletion *tdtA*) | aaatctagaCAGGGACCGCTGTTCTACGTG |
| AB178 (deletion *tdtA*) | aaagaattcCTTGCAGGCCGCTATCACAC |
| AB189 (complementation *tdtA*) | atcaagcttGATCCTTTGCGGGCCGATAC |
| AB190 (complementation *tdtA*) | aaaggatccGACGACGGACGGATCCACATC |
| AB191 (check *tdtA* deletion) | CTGCTGGAGTGAAGGGGGTG |
| AB192 (check *tdtA* deletion) | GATGTGCGCCGCTGGTATG |
| AB193 (check *tdtA* deletion) | CAGGCCATTCCGGACCTG |
| AB194 (check *tdtA* deletion) | CAACGTGCTGCTTGGCCTG |
| AB247 (overexpression *tdtA*) | aaacatatgACGGACCAGGAACTTCTGCA |
| AB248 (overexpression *tdtA*) | aaaaagcttTCATGTCCGCACACGCCCT |
| AB253 (single copy *tdtA*-YFP fusion) | aaaccatggGAAGACCGGGTGCACTACCTG |
| AB254 (single copy *tdtA*-YFP fusion) | aaaactagtTGTCCGCACACGCCCTTG |
| AB255 (multicopy *tdtA*-YFP fusion) | aaaccatggATGACGGACCAGGAACTTCTGCA |
| AB256 (multicopy *tdtA*-YFP fusion) | aaaactatgTCATGTCCGCACACGCCCT |
| AB280 (ICEth13’ marker) | aaatctagaGTGATAGCGGCCTGCAAGCT |
| AB281 (ICEth13’ marker) | aaagaattcGAGCCTCCAGCAGCGGATAG |
| nrcE_fw (NAR1 specific) | GATGGCCTTGCCCTCGGGCCT |
| nrcE_rev (NAR1 specific) | GCTTCTCCACCAGAAACCGGT |
| TTP0220_fw (HB27 specific) | CGGCTGGACCCCA |
| TTP0220_rev (HB27 specific) | CTACTCCGCGAGAAGC |
| TTHA771_fw (HB8 specific) | CCTTGGGGATCCTCTACTAC |
| TTHA771_rev (HB8 specific) | CCCGTATCCCAAGATCCAG |
| tdtA_fw (presence of *tdtA*) | GAGTTATTGGCCGCGCTTC |
| tdtA_rev (presence of *tdtA*) | CATGCGGGTGCTCAGGTG |
| TTC1088_fw (HB27 specific) | GGGCATGAAGGTCCTGA |
| TTC1088_rev (HB27 specific) | CCAAGTTGCCGTCCACC |
